# Supplementary material for: R-loop resolution promotes co-transcriptional chromatin silencing
Source: Nat Commun. 2021 Mar 19;12:1790. doi: 10.1038/s41467-021-22083-6 (PMC7979926; doi:10.1038/s41467-021-22083-6)
Supplement: Supplementary file 1 — Supplementary Information [file 41467_2021_22083_MOESM1_ESM.pdf]

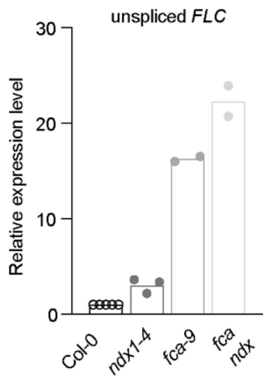

Supplementary Fig. 1 *ndx fca* double mutant shows similar *FLC* expression level to *fca-9*. Unspliced *FLC* relative to *UBC* in various genetic backgrounds, normalized to wild-type Col-0. Data are mean of two to three biological replicates. Source data are provided as a Source Data file.

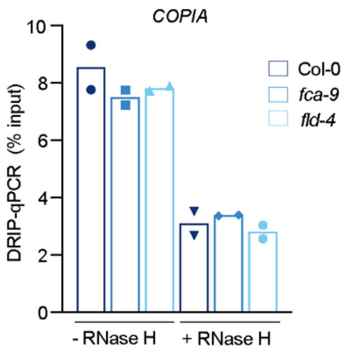

Supplementary Fig. 2 *fca-9* does not affect an R-loop at a *COPIA* element. DRIP-qPCR analysing a GC-rich region over a *COPIA* transposon, with and without RNase H treatment. Data are mean of two biological replicates. Source data are provided as a Source Data file.

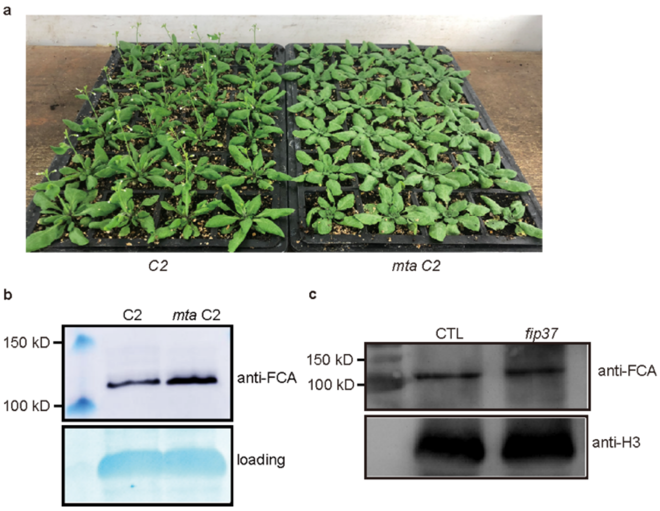

Supplementary Fig. 3 MTA and FIP37 are required for FCA-mediated *FLC* repression, without affecting FCA protein levels. a, A photograph showing flowering phenotype of C2 and *mta* C2 plants 25 days after germination in long day conditions. **b**, FCAγ protein level determined by western blot analysis in C2 and *mta* C2. Loading, Coomassie Blue staining. Representative image from two independent repeats, each includes two biological replicates. **c**, FCA protein level determined by western blot analysis in *fip37* mutant and corresponding control. H3, endogenous control. Representative image from two independent repeats. Full scans for b and c are provided as a Source Data file.

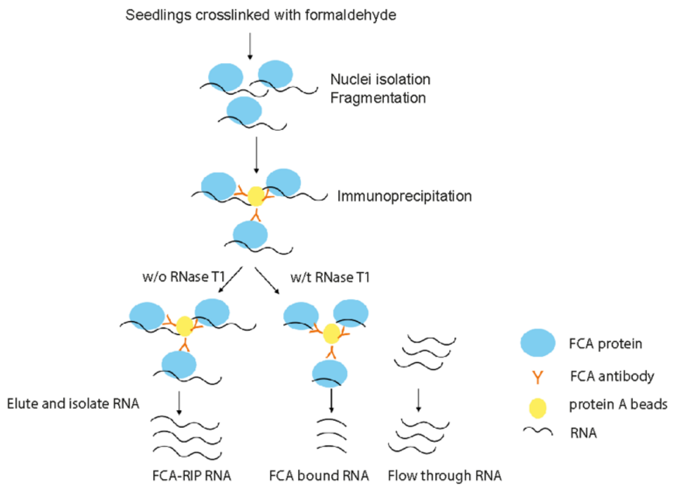

Supplementary Fig. 4 Schematic diagram illustrating steps for detecting m^{6}A enrichment after FCA-RIP.

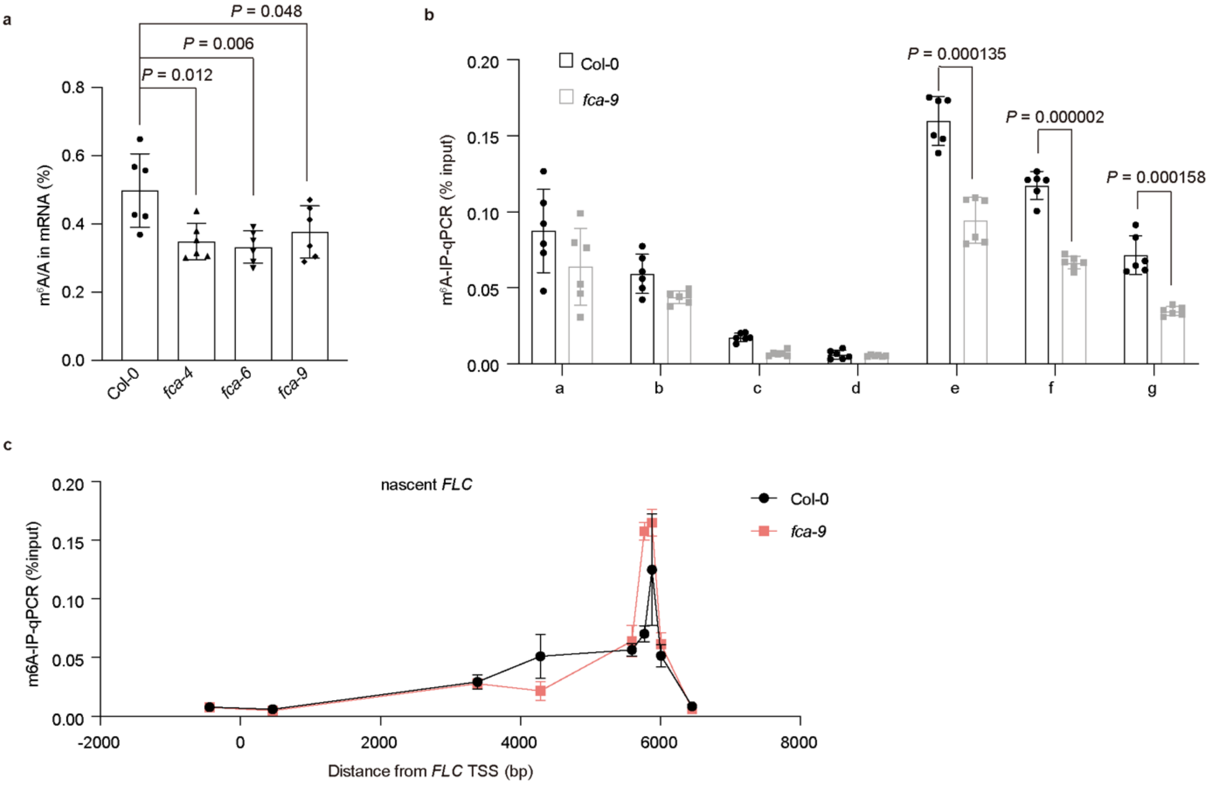

Supplementary Fig. 5 m^{6}A modification is decreased in *fca-9*, both globally and on the *COOLAIR* transcript. a, The m^{6}A-to-A ratio of mRNA determined by LC-MS/MS in wild-type Col-0 and various *fca* alleles. Data are mean ± s.d. from six biological replicates, *P* value from two-tailed *t*-test. **b**, m^{6}A-IP-qPCR analysing m^{6}A enrichment on *COOLAIR* in wild-type and *fca-9*. The x axis represents the amplicons in the qPCR analysis, of which corresponded positions are shown on the schematic in Fig. 2d. Data are mean ± s.d. from six biological replicates, Two-tailed *P* value from multiple *t*-test corrected by Holm-Sidak method. **c,** m^{6}A-IP-qPCR analysing m^{6}A enrichment on *FLC* in wild-type and *fca-9*. The number on x axis is the distance to *FLC* TSS. Data are mean ± s.d. from six biological replicates. Source data are provided as a Source Data file.

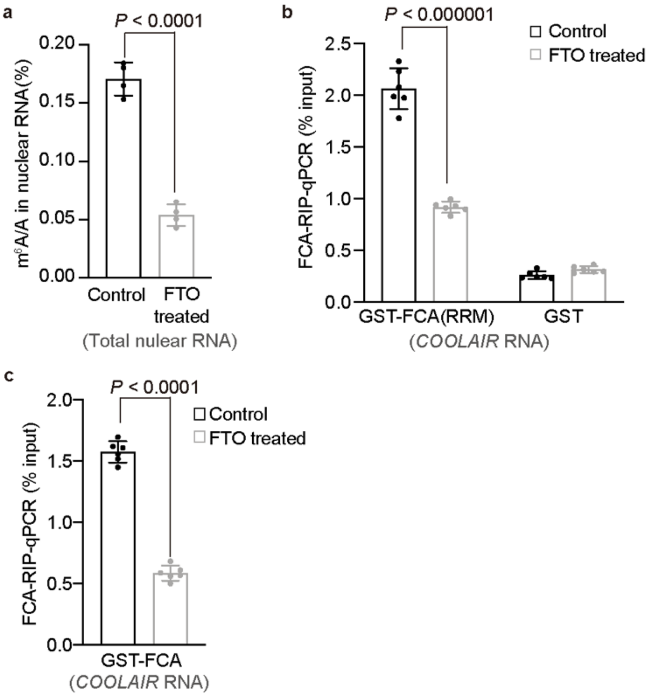

Supplementary Fig. 6 *E. coli* expressed GST-FCA (RRM) and GST-FCA bind with *COOLAIR*, in an m^6A dependent manner. a, LC-MS/MS determined m^6A-to-A ratio on total nuclear RNA, with and without FTO treatment. Data are mean ± s.d. from four biological replicates, *P* value from two-tailed *t*-test. **b,** FCA-RIP-qPCR analysing recombinant GST-FCA (RRM) binding affinity with *COOLAIR* transcript, with and without FTO treatment. GST-only, negative control. Data are mean ± s.d. from six biological replicates, *P* value from two-tailed *t*-test. **c**, FCA-RIP-qPCR analysing recombinant GST-FCA binding affinity with *COOLAIR* transcript with and without FTO treatment. Data are mean ± s.d. from six biological replicates, *P* value from two-tailed *t*-test. Source data are provided as a Source Data file.

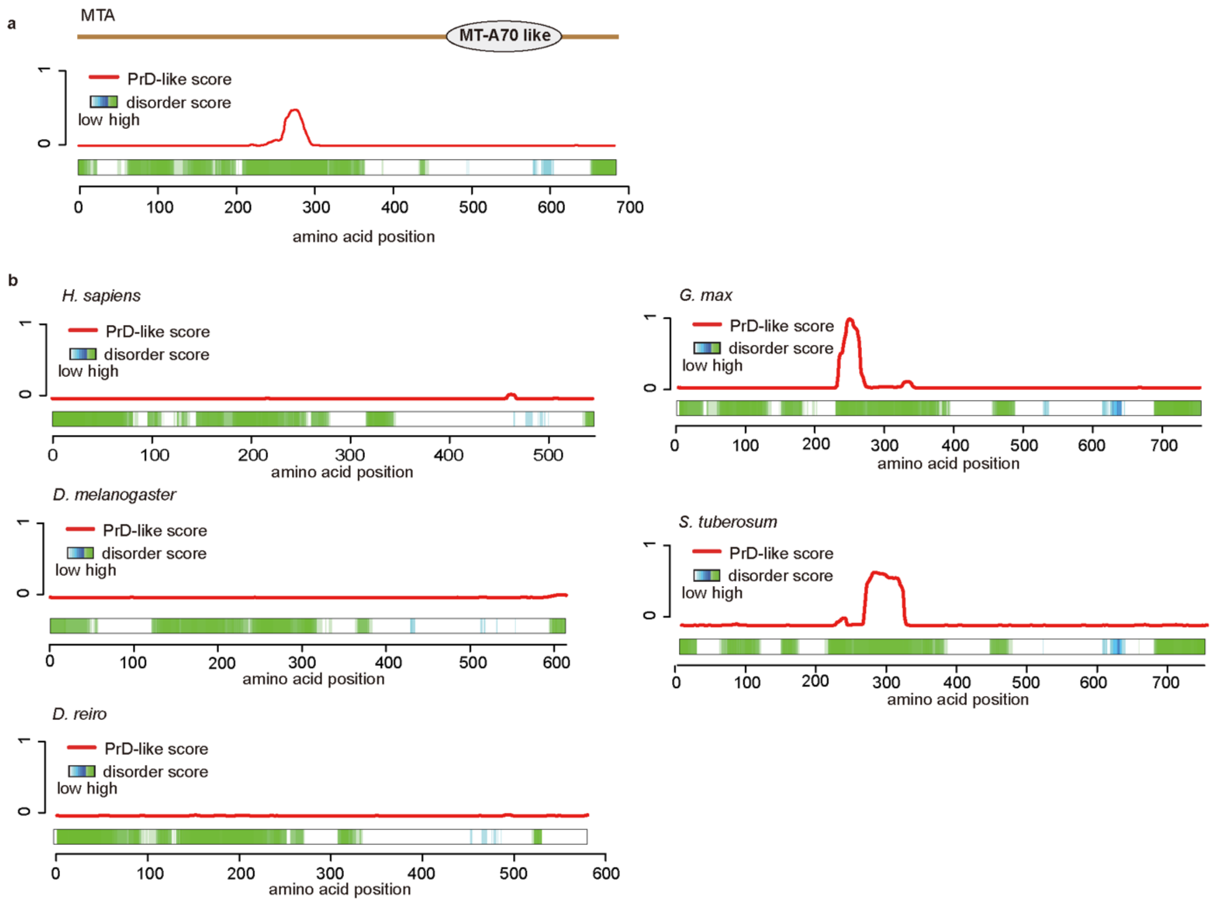

Supplementary Fig. 7 MTA and its orthologs are predicted to be highly disordered. a, Top, the schematic of MTA protein with annotated functional domain. Bottom, predictions of PrLDs and disordered regions by PLAAC[1] and D^{2}P^2 algorithms[2]. **b**, Predictions of PrLDs and disordered regions of MTA orthologs in various organisms by PLAAC and D^{2}P^2 algorithms.

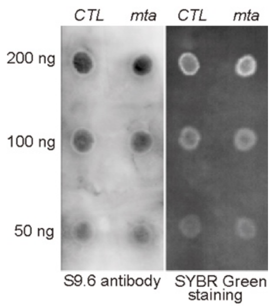

Supplementary Fig. 8 Global R-loop levels do not change in the *mta* mutant. S9.6 dot blot showing global R-loop levels in seedlings in *mta* mutant and corresponding control line (CTL). 50 ng, 100 ng and 200 ng are spotted DNA content. Representative image from two independent repeats. Source data are provided as a source data file.

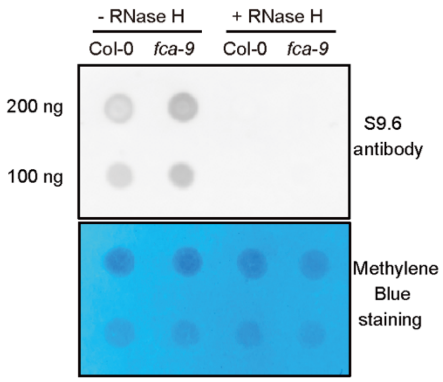

Supplementary Fig. 9 Global R-loop levels accumulate in *fca-9*. S9.6 dot blot showing global R-loop levels in Col-0 and *fca-9*, with and without RNase H treatment. 100 ng and 200 ng are spotted DNA content. Representative image from two independent repeats. Source data are provided as a source data file.

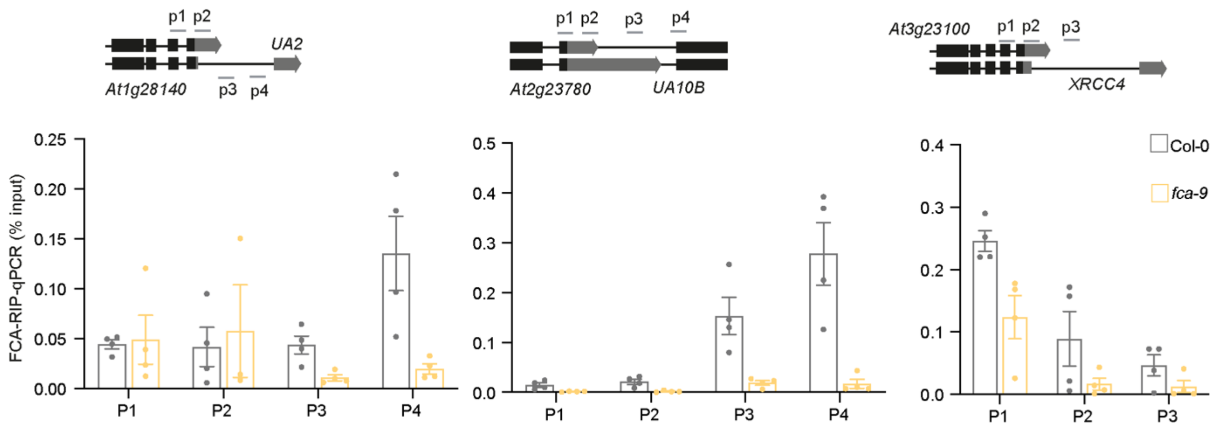

Supplementary Fig. 10 FCA binds to *UA* transcripts. FCA-RIP-qPCR analysing FCA enrichment on the transcripts of *UA*s in wild-type Col-0 and *fca-9*. The x axis represents the amplicons in the qPCR analysis, with positions shown in the schematic. Data are mean ± s.e.m. from four biological replicates. Source data are provided as a Source Data file.

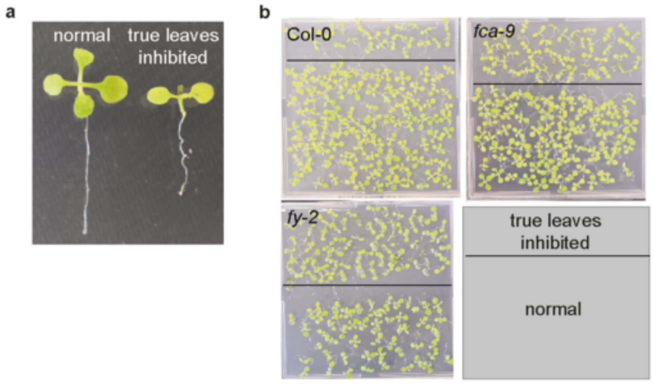

Supplementary Fig. 11 Mutants *fca-9* and *fy-2* are hypersensitive to Bleomycin. a, Representative images of seedlings after bleomycin treatment and recovery (9-day-old seedling), either scored as normal or inhibited as indicated. **b,** After bleomycin treatment and recovery, there are two groups of plants -normal or inhibited true leaf growth, divided by a line shown on the image.

Supplementary Table. 1 List of proteins identified by FCA crosslinked immunoprecipitation.

Identified Protein	Accession No.	Molecular Mass (kDa)	Annotation	No. of Matched Unique Peptides			Sequence Coverage (%)		
				IP1	IP2	IP3	IP1	IP2	IP3
FCA	AT4G16280	82 kDa	RNA binding protein	42	43	40	46	44	39
FY	AT5G13480	72 kDa	3' end processing factor	8	9	10	19	23	22
MTA	AT4G10760	77 kDa	RNA N6-methyladenosine methyltransferase	4	6	13	8	13	27
MTB	AT4G09980	106 kDa	RNA N6-methyladenosine methyltransferase	3	7	9	5	9	16
FIP37	AT3G54170	37 kDa	RNA N6-methyladenosine methyltransferase	0	4	5	0	18	20
HEN2	AT2G06990	112 kDa	putative DExH-box RNA helicase	8	13	13	13	17	16
RH40	AT3G06480	120 kDa	DEAD box RNA helicase family protein	7	10	6	15	23	14
RH14	AT3G01540	68 kDa	DEAD box RNA helicase 1	14	15	22	32	33	36
RH11	AT3G58510	66 kDa	DEA(D/H)-box RNA helicase family protein	4	4	5	18	20	22

Supplementary Table. 2 List of primers used in this study.

Primers used for H3K4me1 CHIP		
**Primer position**	**Sequence 5'-3'**	**Note**
FLC_-392_F<br>FLC_-249_R	ACTATGTAGGCACGACTTTGGTAAC<br>TGCAGAAAGAACCTCCACTCTAC	
FLC_-49_F<br>FLC_58_R	GCCCGACGAAGAAAAAGTAG<br>TTCAAGTCGCCGGAGATACT	
FLC_581_F<br>FLC_672_R	TTTTGTTCATTTCTCTCTCT<br>AAACTTCACTCAACAACATC	
FLC_1533_F<br>FLC_1670_R	TTGACAATCCACAACCTCAATC<br>TCAATTTCCTAGAGGCACCAA	
FLC_2465_F<br>FLC_2579_R	AGTTTGGCTTCCTCATACTTATGG<br>CAATGAACCTTGAGGACAAGG	
FLC_3197_F<br>FLC_3353_R	GGGGCTGCGTTTACATTTTA<br>GTGATAGCGCTGGCTTTGAT	
FLC_3643_F<br>FLC_3752_R	TGAAATGTTACGAATACTAGCGTGT<br>GGATCAAAACTACTAGCTAACCCTTG	
FLC_5030_F<br>FLC_5153_R	CCGGTTGTTGGACATAACTAGG<br>CCAAACCCAGACTTAACCAGAC	
FLC_5672_F<br>FLC_5757_R	CCTGCTGGACAAATCTCCGA<br>GGATTTTGATTTCAACCGCCGA	
FLC_5970_F<br>FLC_6088_R	CGTGTGAGAATTGCATCGAG<br>AAAAACGCGCAGAGAGAGAG	
**Primers used for DRIP-qPCR**		
**Primer position**	**Sequence 5'-3'**	**Note**
FLC_4322_F<br>FLC_4469_R	AGAACAACCGTGCTGCTTTT<br>TGTGTGCAAGCTCGTTAAGC	
FLC_5030_F<br>FLC_5153_R	CCGGTTGTTGGACATAACTAGG<br>CCAAACCCAGACTTAACCAGAC	
FLC_5342_F<br>FLC_5411_R	TTTTTGTTATGGTTAGGTTTGGA<br>AGTAGCACTACTTCTAGACACTTGGA	
FLC_5472_F<br>FLC_5600_R	GCTTCCAAACTTAAAAGCTTAAACA<br>TCTTTTTGTCTTCTATCCAAGGAAT	
FLC_5648_F<br>FLC_5727_R	TAATCATCATGTGGGAGCAG<br>GGAGAGTCACCGGAAGATTG	
FLC_5730_F<br>FLC_5814_R	CACCTTAAATCGGCGGTTG<br>TACAAACGCTCGCCCTTATC	
FLC_5970_F<br>FLC_6088_R	CGTGTGAGAATTGCATCGAG<br>AAAAACGCGCAGAGAGAGAG	
FLC_6768_F<br>FLC_6838_R	TTGTAAAGTCCGATGGAGACG<br>ACTCGGCGAGAAAGTTTGTG	
COPIA_F<br>COPIA_R	TGACGAAGAGCGTACCTGTG<br>CTTGTTTGTCTTCCCCGTGT	
UA2_DRIP_F1<br>UA2_DRIP_R1	AGTTCTTCTTGGGCATTTGG<br>TTTAGAGAAACGACGCAGCA	p1
UA2_DRIP_F2<br>UA2_DRIP_R2	GCTTTCATAGAGGTCTTTGCTATTT<br>AGAAAGGGACATGAACGTGA	p2

UA2_DRIP_F3<br>UA2_DRIP_R3	AAATGTCCATTCCCTGATTGA<br>TTTAAACCGGGTCCAAATCC	p3
UA2_DRIP_F4<br>UA2_DRIP_R4	CATGTCCGACCGTGTTACTG<br>TGGTTGAGTTCATAATTGTAGGAG	p4
UA10B_DRIP_F1<br>UA10B_DRIP_R1	AACCGACCAACAGGTCAAAG<br>AATTCCACCCATCAAACCAA	p1
UA10B_DRIP_F2<br>UA10B_DRIP_R2	TTCAACACCGGGTTATCCTT<br>TGCATCACTTTGGTTTCCTC	p2
UA10B_DRIP_F3<br>UA10B_DRIP_R3	TGTGAAATAAATGGTGCGTGT<br>GATTCAACGTCTGTTCTTCGTTT	p3
XRCC4_DRIP_F1<br>XRCC4_DRIP_R1	TGGACGTTTGAGAAGGAAGG<br>TCAAACATCACAACAAATCCTCA	p1
XRCC4_DRIP_F2<br>XRCC4_DRIP_R2	GATGTCTAGCGCAAGGTGAA<br>TTTAAGCAAGAACAAACCACGA	p2
XRCC4_DRIP_F3<br>XRCC4_DRIP_R3	AGCGAGGAAGAAGCCTCAA<br>TGGAATGGAAACTCTGACCTG	p3
XRCC4_DRIP_F4<br>XRCC4_DRIP_R4	CATAGACCATCACACGAACCA<br>TGTCGAAACAAGCGCAAA	p4
**Primers used for *in vivo* RIP-qPCR**		
**Primer position**	**Sequence 5’-3’**	**Note**
FLC_-49_F<br>FLC_58_R	GCCCGACGAAGAAAAAGTAG<br>TTCAAGTCGCCGGAGATACT	
FLC_1841_F<br>FLC_1954_R	AGTAGTTTGGCCATGTTGGT<br>TCAGGTGTCTCGACAATTCC	
FLC_3643_F<br>FLC_3752_R	TGAAATGTTACGAATACTAGCGTGT<br>GGATCAAAACTACTAGCTAACCCTTG	
FLC_5030_F<br>FLC_5153_R	CCGGTTGTTGGACATAACTAGG<br>CCAAACCCAGACTTAACCAGAC	
FLC_5472_F<br>FLC_5600_R	GCTTCCAAACTTAAAAGCTTAAACA<br>TCTTTTTGTCTTCTATCCAAGGAAT	
FLC_5648_F<br>FLC_5727_R	TAATCATCATGTGGGAGCAG<br>GGAGAGTCACCGGAAGATTG	
FLC_5808_F<br>FLC_5874_R	GATATGTAATTATTCCGCTGATAAGG<br>TCTTGGCCAAAGAGAGAGTATT	
FLC_5970_F<br>FLC_6088_R	CGTGTGAGAATTGCATCGAG<br>AAAAACGCGCAGAGAGAGAG	
UA2_DRIP_F1<br>UA2_DRIP_R1	AGTTCTTCTTGGGCATTTGG<br>TTTAGAGAAACGACGCAGCA	p1
UA2_DRIP_F2<br>UA2_DRIP_R2	GCTTTCATAGAGGTCTTTGCTATTT<br>AGAAAGGGACATGAACGTGA	p2
UA2_DRIP_F3<br>UA2_DRIP_R3	AAATGTCCATTCCCTGATTGA<br>TTTAAACCGGGTCCAAATCC	p3
UA2_DRIP_F4<br>UA2_DRIP_R4	CATGTCCGACCGTGTTACTG<br>TGGTTGAGTTCATAATTGTAGGAG	p4
UA10B_DRIP_F1<br>UA10B_DRIP_R1	AACCGACCAACAGGTCAAAG<br>AATTCCACCCATCAAACCAA	p1
UA10B_DRIP_F2	TTCAACACCGGGTTATCCTT	p2

UA10B_DRIP_R2	TGCATCACTTTGGTTTCCTC	
UA10B_DRIP_F3<br>UA10B_DRIP_R3	TGTGAAATAAATGGTGCGTGT<br>GATTCAACGTCTGTTCTTCGTTT	p3
UA10B_DRIP_F4<br>UA10B_DRIP_R4	GAGCATTCTTGTGCGAGACTAA<br>AGAACAAGAATCGCGGAGTG	p4
XRCC4_DRIP_F1<br>XRCC4_DRIP_R1	TGGACGTTTGAGAAGGAAGG<br>TCAAACATCACAACAAATCCTCA	p1
XRCC4_DRIP_F2<br>XRCC4_DRIP_R2	GATGTCTAGCGCAAGGTGAA<br>TTTAAGCAAGAACAAACCACGA	p2
XRCC4_DRIP_F3<br>XRCC4_DRIP_R3	CATAGACCATCACACGAACCA<br>TGTCGAAACAAGCGCAAA	p3
**Primers used for m^{6}A-IP-qPCR**		
**Primer position**	**Sequence 5'-3'**	**Note**
a_F<br>a_R	GGAAGAACAATGTCGTGAAGAA<br>GCACGCATCAGATCGTATCA	a
b_F<br>b_R	AGCCTTTTAGAACGTGGAACC<br>TCTTCCATAGAAGGAAGCGACT	b
c_F<br>c_R	GTCATTTTCAATCTGCCGAAA<br>AGCAGTAGCACATCTGAATTTCC	c
d_F<br>d_R	AGAACAACCGTGCTGCTTTT<br>TGTGTGCAAGCTCGTTAAGC	d
e_F<br>e_R	TGGTTGTTATTTGGTGGTGTG<br>ATCTCCATCTCAGCTTCTGCTC	e
f_F<br>f_R	CACCTTAAATCGGCGGTTG<br>TACAAACGCTCGCCCTTATC	f
g_F<br>g_R	CGTGTGAGAATTGCATCGAG<br>AAAAACGCGCAGAGAGAGAG	g
**Primers used for expression analysis**		
**Primer position**	**Sequence 5'-3'**	**Note**
UBC_F<br>UBC_R	CTGCGACTCAGGGAATCTTCTAA<br>TTGTGCCATTGAATTGAACCC	for Reverse transcription(RT)
FLC_spliced_F<br>FLC_spliced_R	AGCCAAGAAGACCGAACTCA<br>TTTGTCCAGCAGGTGACATC	for RT
FLC unspliced_F<br>FLC unspliced_R	CGCAATTTTCATAGCCCTTG<br>CTTTGTAATCAAAGGTGGAGAGC	for RT
set2_F<br>set2_R	TCATCATGTGGGAGCAGAAG<br>TCTCACACGAATAAGGTGGCTA	proximal *COOLAIR*
set1_LP	TGGTTGTTATTTGGTGGTGTG	for RT
set4_F<br>set4_R	GTATCTCCGGCGACTTGAAC<br>GGATGCGTCACAGAGAACAG	distal *COOLAIR*
FLC-158_F	GCCCGACGAAGAAAAAGTAG	for RT
**Primers used for genotyping**		
**Primer position**	**Sequence 5'-3'**	**Note**
fca-9_dCAPS_F	TGTTGAGATGGTGAAACTGTG	
fca-9_dCAPS_R	TCTTTGGCTCAGCAAACC	
p745<br>ndx1-4_GT_F<br>ndx1-4_GT_R	AACGTCCGCAATGTGTTATTAAGTTGTC<br>TTGAGGTGTGACTGATTGCC<br>TTGAGGTGTGACTGATTGCC	

Supplementary References

1. Lancaster, A. K., Nutter-Upham, A., Lindquist, S. & King, O. D. PLAAC: a web and command-line application to identify proteins with prion-like amino acid composition. *Bioinformatics* **30**, 2501–2502 (2014).

2. Oates, M. E. et al. D2P2: Database of disordered protein predictions. *Nucleic Acids Res.* **41**, 508–516 (2013).
