# Supplementary material for: R-loop resolution promotes co-transcriptional chromatin silencing
Source: Nat Commun. 2021 Mar 19;12:1790. doi: 10.1038/s41467-021-22083-6 (PMC7979926; doi:10.1038/s41467-021-22083-6)
Supplement: Supplementary file 3 — Reporting Summary [file 41467_2021_22083_MOESM3_ESM.pdf]

nature research

Corresponding author(s): Caroline Dean
Guifang Jia

Last updated by author(s): Jan 27, 2021

Reporting Summary

Nature Research wishes to improve the reproducibility of the work that we publish. This form provides structure for consistency and transparency in reporting. For further information on Nature Research policies, see our Editorial Policies and the Editorial Policy Checklist.

Statistics

For all statistical analyses, confirm that the following items are present in the figure legend, table legend, main text, or Methods section.

n/a	Confirmed	
☐	☒	The exact sample size (*n*) for each experimental group/condition, given as a discrete number and unit of measurement
☐	☒	A statement on whether measurements were taken from distinct samples or whether the same sample was measured repeatedly
☐	☒	The statistical test(s) used AND whether they are one- or two-sided<br>*Only common tests should be described solely by name; describe more complex techniques in the Methods section.*
☒	☐	A description of all covariates tested
☐	☒	A description of any assumptions or corrections, such as tests of normality and adjustment for multiple comparisons
☐	☒	A full description of the statistical parameters including central tendency (e.g. means) or other basic estimates (e.g. regression coefficient) AND variation (e.g. standard deviation) or associated estimates of uncertainty (e.g. confidence intervals)
☐	☒	For null hypothesis testing, the test statistic (e.g. *F*, *t*, *r*) with confidence intervals, effect sizes, degrees of freedom and *P* value noted<br>*Give P values as exact values whenever suitable.*
☒	☐	For Bayesian analysis, information on the choice of priors and Markov chain Monte Carlo settings
☒	☐	For hierarchical and complex designs, identification of the appropriate level for tests and full reporting of outcomes
☒	☐	Estimates of effect sizes (e.g. Cohen's *d*, Pearson's *r*), indicating how they were calculated

Our web collection on statistics for biologists contains articles on many of the points above.

Software and code

Policy information about availability of computer code

Data collection	Raw Cp value of qPCR was collected with LightCycler® 480 Software. Microscope images were collected with ZEN Black software on Zeiss LSM780 confocal microscope.
Data analysis	GraphPad Prism version 8.4.3 was used for statistic analysis. Image J for MacOS 2.0.0-rc-69/1.52p was used for image analysis. PLAAC (http://plaac.wi.mit.edu/) and D2P2 (http://d2p2.pro/) algorithms were used for predictions of protein disordered regions.

For manuscripts utilizing custom algorithms or software that are central to the research but not yet described in published literature, software must be made available to editors and reviewers. We strongly encourage code deposition in a community repository (e.g. GitHub). See the Nature Research guidelines for submitting code & software for further information.

Data

Policy information about availability of data

All manuscripts must include a data availability statement. This statement should provide the following information, where applicable:

- Accession codes, unique identifiers, or web links for publicly available datasets
- A list of figures that have associated raw data
- A description of any restrictions on data availability

Source data for figures are provided in the source data file with this paper. Raw data and processed images are available from https://figshare.com/s/955154d8d889349af223 under folders Figure 3c and Figure 3d. All relevant data are available from the corresponding authors upon reasonable request.

April 2020

Field-specific reporting

Please select the one below that is the best fit for your research. If you are not sure, read the appropriate sections before making your selection.

☒ Life sciences ☐ Behavioural & social sciences ☐ Ecological, evolutionary & environmental sciences

For a reference copy of the document with all sections, see nature.com/documents/nr-reporting-summary-flat.pdf

Life sciences study design

All studies must disclose on these points even when the disclosure is negative.

Sample size	No statistical approach was used to predetermine sample size. Sample size is specified in each figure legend. Sample sizes were determined based on similar experiments on previous publications.
Data exclusions	In Figure 3c, 3d, when using image J for nuclear body analysis, we excluded one pixel sized particle as we can't differentiate these are real particles or backgrounds due to diffraction.
Replication	Primary findings were reproduced in several independent experiments or several biological replicates with technical repeats, as indicated in the figure legends. For experiments with reproducible data which were not included in this study, the replications were successful.
Randomization	Hundreds or thousands of seedlings grown on one or more plates were randomly collected for each replicate for molecular biology experiments. In microscopy analysis, images were taken from 30 individuals which were grown under the same condition. Data were collected from individuals with the same genetic background (homozygous) in each group.
Blinding	In microscopy analysis, the same settings were used in data collection and data analysis. In all the experiments, absolute blinding is not possible as the investigators who performed experiments also analysed the data.

Reporting for specific materials, systems and methods

We require information from authors about some types of materials, experimental systems and methods used in many studies. Here, indicate whether each material, system or method listed is relevant to your study. If you are not sure if a list item applies to your research, read the appropriate section before selecting a response.

Materials & experimental systems

n/a	Involved in the study	
☐	☒	Antibodies
☒	☐	Eukaryotic cell lines
☒	☐	Palaeontology and archaeology
☒	☐	Animals and other organisms
☒	☐	Human research participants
☒	☐	Clinical data
☒	☐	Dual use research of concern

Methods

n/a	Involved in the study	
☒	☐	ChIP-seq
☒	☐	Flow cytometry
☒	☐	MRI-based neuroimaging

Antibodies

Antibodies used

Anti-DNA-RNA Hybrid [S9.6] Antibody, Kerafast, ENH001;
FCA, homemade (western blot 1:5,000 dilution)
m6A antibody, Synaptic Systems, Cat. No. 202003;
Anti-Histone H3 antibody, Abcam, ab1791;
Anti-Histone H3K4me1 antibody, Abcam, ab8895
Amersham ECL Mouse IgG HRP-linked whole Ab (from sheep), GE Healthcare, NA931; (1:20,000 dilution)
Amersham ECL Rabbit IgG HRP-linked whole Ab (from donkey), GE Healthcare, NA934; (1:10,000 dilution)
protein G agarose, Invitrogen, 15920010
Dynabeads protein A, Invitrogen, 10002D

Validation

S9.6 antibody
Signal was significantly decreased after RNase H treatment which specifically digests RNA/DNA hrbrids (Fig1a, 1b). This antibody was shown to bind dsRNA with low affinity (Hartono et al., 2018). We pretreated samples with RNase A which digests RNAs in DRIP and dot blot.
FCA antibody
The validation has been previously described (Macknight et al., 2002).
m6A antibody
The manufacture states it is applicable to IP and is specific for N6-methyladenosine (m6A) with some cross-reactivity to m6Am. In

Arabidopsis, this antibody was previously validated for IP (Shen et al., 2016; Duan et al., 2017) and the cross-reactivity to m6Am is not a problem as m6Am is not detected in Arabidopsis poly(A)+ RNA (Duan et al., 2017).
H3 antibody
The manufacture states it is applicable to WB as nuclear loading control in many species including Arabidopsis.
H3K4me1 antibody
The manufacture states it is a ChIP-grade antibody and works with plants.

Hartono, SR et al. The Affinity of the S9.6 Antibody for Double-Stranded RNAs Impacts the Accurate Mapping of R-Loops in Fission Yeast. JMB, 430, 272-284 (2018)
Mackight, R et al. Functional Significance of the Alternative Transcript Processing of the Arabidopsis Floral Promoter FCA. The Plant Cell, 14, 877–888 (2002)
Shen, L. et al. N -Methyladenosine RNA Modification Regulates Shoot Stem Cell Fate in Arabidopsis. Dev. Cell 38, 186–200 (2016).
Duan, H. C. et al. ALKBH10B is an RNA N -methyladenosine demethylase affecting arabidopsis floral transition. Plant Cell 29, 2995–3011 (2017).
